# Emotion Regulation Difficulties and Sexual Motivation Associated with Faking Orgasm among Hungarian Women

**DOI:** 10.1080/19317611.2025.2514770

**Published:** 2025-06-08

**Authors:** Norbert Meskó, Edit Csányi, Orsolya Inhóf, András N. Zsidó

**Affiliations:** Institue of Psychology, University of Pécs, Pécs, Hungary

**Keywords:** Faking orgasm, emotion regulation, sexual motivation, women’s sexual behavior, sexual communication

## Abstract

**Objectives:**

Faking orgasm is a common yet psychologically complex behavior among women, shaped by both emotional vulnerabilities and sexual motivations. This study examined the psychological correlates of faking orgasm in two sexual contexts—vaginal intercourse and oral sex—focusing on difficulties in emotion regulation and sexual motivation.

**Method:**

A sample of 425 Hungarian women completed self-report measures assessing six facets of emotion regulation (Difficulties in Emotion Regulation Scale), three types of sexual motivation (Hungarian Short Form of the Reasons for Having Sex Questionnaire), and four motives for faking orgasm in each context (Faking Orgasm Scale). We used a dual analytic approach combining network analysis and multiple linear regressions to explore and predict context-specific patterns.

**Results:**

Faking orgasm was associated with emotion regulation difficulties—especially non-acceptance of emotions, impulse control problems, and lack of emotional clarity—and with sexual motivations related to coping, personal goals, and partner-focused concerns. These associations were stronger and more interconnected in vaginal intercourse than in oral sex. Network centrality analyses identified coping-related motivation and emotion regulation deficits as key variables.

**Conclusions:**

Faking orgasm may serve as a strategic emotion regulation behavior embedded in relational dynamics and sociocultural scripts. The findings highlight the importance of addressing emotional literacy, sexual communication, and relational expectations in clinical and educational contexts. Future research should investigate real-time affective processes and cultural norms that shape women’s sexual behavior.

## Introduction

### Understanding female orgasm and the phenomenon of faking

Female orgasm has been described as a multifaceted biopsychosocial phenomenon involving physiological, psychological, and relational processes that serve diverse functions in both sexual and relational contexts (Mah & Binik, 2001). Although orgasm contributes to women’s sexual satisfaction, it is only one of several relevant factors, including emotional intimacy, sexual communication, and the relational context (Brody & Costa, 2009). Orgasms occur more frequently in committed relationships than in casual sexual encounters (Armstrong et al. 2012), suggesting that relational factors shape female orgasmic experiences. Qualitative research highlights factors that enhance women’s ability to achieve orgasm, including strong interpersonal connections, participation in non-penetrative sexual activities, and positive body image (Fahs, 2014; Muehlenhard & Shippee, 2010).

Faking orgasm, explicitly documented by Masters and Johnson (1966), refers to deliberately imitating outward signs and sensations of sexual climax without experiencing the underlying physiological and psychological processes. Early research emphasized the relational and interpersonal dynamics underlying women’s motivations for fake orgasms, highlighting how this behavior reflects broader relationship enhancement strategies (Darling & Davidson, 1986). Scholars have examined this phenomenon through frameworks such as evolutionary psychology, sociocultural paradigms, and interpersonal relationship dynamics (Cooper et al., 2014; Hevesi et al., 2022; Láng et al., 2020; McCoy et al., 2015). Faking orgasm occurs in various sexual contexts, including vaginal intercourse, oral sex, and other partnered sexual activities, and sexual motivations may differ across these contexts (Harris et al., 2016; Muehlenhard & Shippee, 2010). In line with Cooper’s (2014) use of the term ‘sexual intercourse’ we refer to this behavior more precisely as ‘vaginal intercourse’ throughout this study.

It is crucial to distinguish between faking orgasm and orgasm difficulties. Orgasm difficulties refer to persistent challenges in reaching orgasm, while faking orgasm is a deliberate act serving psychological or relational functions independent of physiological capacity (Herbenick et al., 2019). This behavior, reported by a significant proportion of women, is associated with psychological and relational factors such as partner expectations and emotional regulation strategies (Muehlenhard & Shippee, 2010).

Understanding faking orgasm is essential because although it may serve adaptive functions such as avoiding conflict, preserving a partner’s self-esteem, or maintaining harmony during sexual interactions (Wiederman, 1997; Herbenick et al., 2019), it can also indicate underlying emotional or relational difficulties (Fahs, 2014; Thomas et al., 2017). The orgasmic response in women is a complex psychophysiological process that involves sensory, cognitive, and emotional components (Laan & Both, 2008). While orgasms are commonly linked to sexual pleasure and relational bonding, their absence, particularly in contexts with strong expectations or relational demands, may lead to dissatisfaction, distress, or relational tension (Basson & Gilks, 2018; Brotto et al., 2016). These interpersonal and emotional layers of orgasmic experiences underscore the importance of examining faking orgasm as it offers insight into how individuals manage sexual communication, expectations, and intimacy.

### The role of emotion regulation difficulties in faking orgasm

Difficulties in emotion regulation refer to impairments in identifying, understanding, managing, and responding adaptively to emotional states. According to Gratz and Roemer (2004), these difficulties can manifest as the non-acceptance of emotional responses, impulse control problems, and limited access to strategies that help achieve personal goals despite negative affect. Emotional dysregulation may, therefore, lead individuals to engage in behaviors that serve to manage emotional discomfort, including those occurring in intimate and sexual contexts (Aldao et al., 2010; Gross & John, 2003).

Surprisingly, despite the wide range of emotional experiences that may occur during sex, research examining the role of emotion regulation in normative sexual behavior remains limited. Most prior studies have focused on clinical or high-risk populations (e.g., Rellini et al., 2010; Noll et al., 2011), leaving a gap in understanding how emotion regulation processes influence common, non-pathological sexual behaviors, such as faking orgasm. Moura et al. (2020) have called attention to this gap, noting that everyday sexual functioning may also be shaped by underlying affective regulation strategies. In support of this, Mah and Binik (2001) suggested that women’s sexual responses are closely tied to psychological adaptation, underscoring the interplay between emotional states and sexual outcomes.

On average, women report a broader range of sex-related emotional responses and are more likely to experience negative emotions such as fear, anxiety, or guilt during sexual activity, all of which have been associated with reduced sexual satisfaction and functioning (Dosch et al., 2016; Purdon & Holdaway, 2006; Mark & Murray, 2012). They may also attribute greater emotional or relational meaning to sexual encounters than to men, although this varies across individuals and contexts (Brom et al., 2015, 2016; Everaerd et al., 2006). Qualitative accounts suggest that, for some women, faking orgasm may function as an interpersonal strategy to avoid disappointing a partner, reduce relational tension, or end a dissatisfying encounter without confrontation (Cooper et al., 2014; Muehlenhard & Shippee, 2010).

Furthermore, recent studies have shown that difficulties in emotion regulation are associated with various aspects of sexual functioning and satisfaction, even outside clinical contexts (Fischer et al., 2024; Pepping et al., 2018; Viana-Sousa et al., 2023). These findings suggest that emotional dysregulation can play a role in shaping how individuals communicate and behave sexually; especially in situations where relational expectations are high or emotional needs conflict with sexual desires.

To date, only one study has explicitly investigated the link between emotion regulation and fake orgasm. Cooper (2014) found that women with more pronounced difficulties in emotion regulation, particularly in emotional clarity and acceptance, were more likely to report frequent faking of orgasm. These findings support the idea that faking orgasm can be understood as a regulatory strategy deployed under emotional strain, functioning to maintain a sense of control, avoid distress, or preserve harmony in a sexual relationship. This interpretation is consistent with earlier research indicating that women with orgasmic difficulties often report self-blame, emotional suppression, and heightened emotional dependency or worry (Rowland et al., 2018; Tavares et al., 2018).

Taken together, these results underscore the importance of examining how individual differences in emotion regulation contribute to the tendency to fake orgasm, particularly when sexual activity is emotionally or relationally complex. The present study aimed to investigate these links systematically using both network and regression analyses to explore the interplay between the dimensions of emotion regulation and orgasm-faking behaviors.

### Sexual motivation and the strategic use of orgasm faking

Sexual motivation refers to the internal drives, intentions, and desires that lead individuals to engage in sexual activities (Meston & Buss, 2007). These motivations are psychologically complex, and span a wide spectrum of reasons that extend beyond the pursuit of physical pleasure. While early research identified only a handful of sexual motives, subsequent studies have revealed that people engage in sex for a broad array of reasons, including emotional connections, reassurance, relational maintenance, curiosity, and even avoidance of conflict (Meston & Buss, 2007; Meskó et al., 2022).

Although gender differences in sexual motivation have been consistently documented, with women more frequently citing emotional or relational motives (e.g., love, intimacy, commitment) and men more often mentioning physical gratification or novelty, recent research emphasizes substantial individual variation and overlap across genders (Impett et al., 2005; Petersen & Hyde, 2010). Moreover, sexual motivation is not static; it evolves throughout the lifespan and is influenced by hormonal, psychological, and relational factors (Klusmann, 2002; Toates, 2009).

One particularly relevant dimension of sexual motivation in the context of a faking orgasm is the use of sex as a form of emotional regulation. This coping-related sexual motivation reflects the tendency to engage in sexual behavior, not solely for pleasure or connection, but also as a means to manage internal emotional states or relational tension. For example, women may have sex to reduce anxiety, avoid relational conflict, or alleviate feelings of insecurity and fear of rejection (Cooper, 2014; Pham et al., 2015). In this context, sex can serve as a psychological coping strategy, rather than a purely hedonic or relational act.

Recent studies have empirically demonstrated a connection between coping-related sexual motivation and faking orgasm. In a cluster-analytic study, Cooper (2014) identified emotion regulation profiles associated with the frequency of and motivation for faking orgasm. Similarly, Csányi, Basler, et al. (2024) found that coping-related motives significantly predicted faking orgasm across multiple contexts, including oral sex and vaginal intercourse. These findings suggest that, for many women, faking orgasm may be a behavioral manifestation of underlying affect regulation processes, serving to maintain emotional equilibrium or preserving relational stability under conditions of emotional discomfort.

Furthermore, sexual behavior is embedded in sociocultural norms and expectations. Sexual script theory posits that people internalize cultural narratives on how sexual interactions should unfold (Gagnon & Simon, 1973). Within many heteronormative contexts, women are often expected to prioritize their partners’ satisfaction, avoid confrontation, and maintain harmony within the relationship. These expectations may encourage behaviors such as a faking orgasm, which functions as a strategic response to social pressure or interpersonal dynamics (Lehmiller, 2023; Meskó et al., 2022). In certain situations, orgasm itself may be commodified, with women simulating sexual climax as a means of meeting perceived relational obligations or avoiding difficult conversations (Frith, 2018; Thomas et al., 2017).

Taken together, these perspectives highlight that faking orgasm may be driven not only by situational demands but also by deeper emotional and motivational mechanisms. By integrating emotion regulation difficulties and coping-related sexual motives into a unified framework, researchers can better understand the complex psychological processes underlying this behavior. The present study aimed to explore these intertwined influences, with special attention paid to the distinct roles played by sexual context (vaginal intercourse vs. oral sex), emotional dysregulation, and motivational functions.

### The present study

Building on prior research on the psychological mechanisms underlying faking orgasm, the present study aimed to examine how emotion regulation difficulties and sexual motivations contribute to this behavior in women. While previous studies have emphasized the interpersonal and affective functions of faking orgasm, few have systematically investigated the role of distinct emotion regulation deficits and coping-related sexual motives in a nonclinical context.

We focused on two core psychological domains: (1) emotion regulation difficulties, including non-acceptance of emotions, impulse control problems, and lack of emotional clarity; and (2) sexual motivations, with an emphasis on coping-related motives, that is, engaging in sex to manage negative emotions, reduce relational tension, or prevent rejection.

The behavioral outcome of interest, faking orgasm, was explored across two sexual contexts: oral sex and vaginal intercourse, following the structure of prior research (e.g., Cooper, 2014). This contextual distinction allowed us to examine whether emotional and motivational predictors operate differently across different types of sexual activities.

Given the limited number of studies examining these psychological variables in tandem, and the multivariate nature of their relationships, we employed an exploratory approach using both network analysis and multiple regression modeling.

Accordingly, we posed the following research question:

**RQ1:** How are different facets of emotion regulation difficulties (e.g., non-acceptance of emotions, impulse control, and emotional clarity) associated with various motives for faking orgasm in women?

**RQ2:** To what extent do emotion regulation difficulties and coping-related sexual motivations predict the tendency to fake orgasm during vaginal intercourse and oral sex?

Based on prior findings (Cooper, 2014; Csányi, Basler, et al., 2024), we expected that faking orgasm would be most strongly associated with coping-related sexual motives and difficulties in non-acceptance of emotions, emotional clarity, and impulse control. We also anticipated that these associations might be more pronounced in the context of vaginal intercourse than in oral sex.

## Hypotheses

Based on previous research, we formulate the following hypotheses:

H1: Difficulties in emotion regulation—particularly non-acceptance of emotions, impulse control problems, and lack of emotional clarity—are positively associated with the tendency to fake orgasm.

H2: Coping-related sexual motivation—particularly motives such as partner reassurance, emotional coping, and conflict avoidance—is positively associated with the tendency to fake orgasm, independent of other motivational dimensions (e.g., ego-focused or relationship-focused motives).

While this study did not aim to formally test interaction effects across sexual contexts, we anticipated that the strength of the associations between psychological predictors and orgasm faking might differ between vaginal intercourse and oral sex, with potentially stronger links emerging in the context of vaginal intercourse.

## Method

### Participants

This study was conducted in Hungary using a convenience sample of 425 self-identified women. All participants were assigned female at birth and identified as women. Their age ranged from 18 to 80 years (M = 24.51, SD = 7.74). All participants had previously engaged in sexual intercourse and 41 (9.6%) had at least one child.

The participants’ relationship status varied at the time of the survey: 40.0% were dating, 29.6% cohabiting, 6.8% married, 5.6% engaged in casual sex with multiple partners, and 0.9% reported other arrangements (e.g., friends with benefits). Among them, 353 (83%) were in a sexual relationship at the time of data collection.

Sexual orientation was measured on a 7-point Likert scale ranging from exclusively heterosexual to exclusively homosexual; 85.2% of the respondents identified as exclusively heterosexual. Although the sex of sexual partners was not directly assessed, the orientation data suggested that most partnered sexual activity occurred with male partners.

Participants were asked to reflect on either their current or past relationship(s) in which they had faked an orgasm regardless of their current relationship status. This allowed for the inclusion of broader experiential data and captured retrospective insights into faking behavior. Prior research supports the utility of such retrospective accounts for understanding the relational and psychological dynamics in sexual behavior (Cooper, 2014; Cooper et al., 1998; Muehlenhard & Shippee, 2010).

Among the total sample, 151 women (35.5%) reported a faking orgasm during oral sex and 208 (48.9%) during vaginal intercourse. A total of 184 participants (43.3%) had faked orgasms in both contexts, whereas 123 (28.9%) reported that they had never done so. Participants who never faked orgasm (*N* = 123) were excluded from analyses where faking orgasm was the dependent variable (e.g., regression) but were included in descriptive and correlational analyses.

### Procedure

Participants were recruited online via social media platforms (e.g., Facebook and Instagram) and university mailing lists. The anonymous survey was administered using Qualtrics and took approximately 15–20 minutes to complete. Participation was voluntary and no compensation was offered.

To ensure adequate statistical power, the required sample size was determined using G*Power 3 (Faul et al., 2007) with f = 0.20, power = 0.95, and nine predictors, yielding a minimum of 127 participants. A larger sample size was targeted to ensure robustness in both the regression and network analyses. For the latter, sample sizes between 250 and 350 were generally sufficient to detect moderate sensitivity in edge weights for networks with up to 20 nodes (Constantin & Cramer, 2020).

Informed consent was obtained from all participants before they began the survey. This study was approved by the United Ethical Review Committee for Research in Psychology in Hungary (ref. No. 2022/107) and was not preregistered.

### Measures

Before completing the questionnaires, the participants provided demographic information including age, sex assigned at birth, gender identity, sexual orientation, and current relationship status. They were also asked whether they had ever experienced an orgasm during vaginal intercourse and/or when receiving oral sex. When responding to the Faking Orgasm Scale for Women (FOS; Cooper et al., 2014), participants were instructed to reflect on a current or past relationship in which they had faked orgasm, if applicable.

#### Faking Orgasm Scale for Women (FOS)

The FOS (Cooper et al., 2014; Hungarian adaptation: Csányi, Őry, et al., 2024) is a self-report instrument designed to assess motives for faking orgasm in two distinct sexual contexts: vaginal intercourse (35 items) and oral sex (26 items). Each context-specific set included four subscales capturing motivational dimensions such as enhancing a partner’s experience, avoiding negative consequences, emotion regulation, and reinforcing intimacy. This structure allows for a nuanced understanding of why women fake orgasms in different sexual contexts.

In the present study, the Oral Sex subscales were completed only by participants who reported having a faked orgasm while receiving oral sex (*N* = 151). The subscales evaluate several motives for faking orgasm: (1) Altruistic Deceit (OSAD (partner assurance during oral sex)), which involves simulating orgasm to protect a partner’s feelings (e.g., Because you believe it is important for your partner to feel they can please you); (2) Insecure Avoidance (OSIA (insecurity avoidance during oral sex)), where orgasm is faked to evade feelings of insecurity (e.g., because you feel physically uncomfortable during oral sex); (3) Elevated Arousal (OSEA (emotional avoidance during oral sex)), where the aim is to enhance one’s own sexual arousal (e.g., To turn yourself on); and (4) Fear of Dysfunction (OSFD (fear of displeasing one’s partner during oral sex)), which involves faking orgasm to address worries about being abnormal (e.g., because you suspect something might be physically wrong if you do not orgasm). The McDonald’s ω values for the four measures were .93, .76, .83, and .83, respectively.

The Sexual Intercourse subscales were only completed by those participants who reported having faked orgasm during sexual intercourse (*N* = 208). The subscales evaluate the following motivations for faking orgasm: (1) Altruistic Deceit (SIAD (partner assurance during sexual intercourse)), where individuals pretend to climax to acknowledge their partner’s efforts (e.g., Because you want to reward your partner for their effort); (2) Fear and Insecurity (SIFI (insecurity avoidance during sexual intercourse)), which involves faking orgasm to escape negative feelings related to the sexual experience (e.g., to avoid feeling badly about yourself if you do not have a real orgasm); (3) Elevated Arousal (SIEA (emotional avoidance during sexual intercourse)), where the intention is to boost one’s own sexual excitement (e.g., to increase arousal during sexual intercourse); and (4) Sexual Adjournment (SISA (sexual anxiety during sexual intercourse)), where orgasm is faked to conclude sexual activity (e.g., because you want to go to sleep). The McDonald’s ω values for the four measures were .94, .88, .93, and .80, respectively.

It is important to note that the Faking Orgasm Scale includes separate context-specific subscales for oral sex and vaginal intercourse, each with distinct items. Therefore, no direct statistical comparison was conducted between the two contexts as they represent conceptually and psychometrically distinct constructs.

#### Difficulties in emotion regulation Scale (DERS)

The DERS (Gratz & Roemer, 2004; Hungarian adaptation by Kökönyei et al., 2014) is a self-report scale consisting of 36 questions used to measure emotion dysregulation. The total score provides an overall measure of emotion regulation difficulties, while six specific difficulties are assessed by the following subscales: (1) Non-Acceptance of emotional responses (Non-Acceptance; 6 items; e.g., *When I’m upset, I feel guilty for feeling that way*); (2) Difficulties engaging in goal-directed behavior (Goals; 5 items; e.g., *When I’m upset, I have difficulty getting work done*); (3) Impulse control difficulties (Impulse; 6 items; e.g., *When I’m upset, I become out of control*), which measures difficulties in behavioral control and regulation when experiencing (negative) emotions; (4) Lack of emotional awareness (Awareness; 6 items; e.g., *I pay attention to how I feel* [reverse-scored item]), which assesses one’s tendency to ignore internal affective signals; (5) Limited access to emotion regulation strategies (Strategies; 8 items; *When I’m upset, I believe that there is nothing I can do to make myself feel better*); and (6) Lack of emotional clarity (Clarity; 5 items; *I am confused about how I feel*). Responses to each item are given on a 5-point rating scale ranging from *Almost Never* (1) to *Almost Always* (5). McDonald’s ω values for the Non-Acceptance, Goals, Impulse, Awareness, Strategies, and Clarity subscales were .88, .91, .90, .79, .92, and .90, respectively.

#### Reasons for having sex questionnaire, Hungarian short form (YSEX?-HSF)

The YSEX?-HSF, developed by Meskó et al. (2022), is a self-report tool designed to assess sexual motivation. The Hungarian version was based on the original American YSEX? questionnaire by Meston and Buss (2007) but consists of three main variables rather than four. Although these two questionnaires share similar items, their variable structures differ slightly, highlighting both universal and culturally specific aspects of human sexual motivation. The YSEX?-HSF consists of 73 items organized into three scales: (1) Personal Goal Attainment (e.g., I wanted a new experience), (2) Relational Reasons (e.g., I was in love), and (3) Sex as Coping (e.g., I wanted to save the relationship). Each item is rated on a 5-point scale, from 1 (none of my sexual experiences) to 5 (all of my sexual experiences), with higher scores indicating stronger motivation in each category. McDonald’s ω values for personal goal attainment, relational reasoning, and sex as coping were .92, .90, and .91, respectively.

### Statistical analyses

The primary aim of this study was to examine how psychological variables—emotion regulation difficulties and sexual motivations—predict the tendency to fake orgasm in two distinct sexual contexts: vaginal intercourse and oral sex. To address this, we used a combination of network analysis and multiple linear regression models following an exploratory-confirmatory analytic strategy.

First, we applied a network analysis to explore the partial correlations among the psychological predictors (that is, six DERS subscales and three YSEX?-HSF subscales), and Faking Orgasm Scale subscales. Two separate networks were estimated: one for vaginal intercourse (FOS-SI subscales), and one for oral sex (FOS-OS subscales). This approach allowed us to visualize the structure of associations and identify the central psychological variables within each sexual context.

The DERS subscales include non-acceptance of emotional responses, difficulties in goal-directed behavior, impulse control problems, lack of emotional awareness, limited access to effective regulation strategies, and lack of emotional clarity. The three YSEX?-HSF subscales represented coping-related (coping with emotional distress), self-focused (personal goal attainment), and relationship-focused (partner-related relational goals) sexual motivations.

Network estimation was performed using the graphical Least Absolute Shrinkage and Selection Operator (glasso), with the extended Bayesian Information Criterion (EBIC) and a tuning parameter of 0.5 (Epskamp et al., 2018). Edge weights represent regularized partial correlations, and centrality indices (strength and expected influence) were computed to determine the most influential nodes. The stability of the network metrics was evaluated via nonparametric bootstrapping (1,000 iterations), and the correlation stability (CS) coefficient was used to assess reliability.

Next, to formally test our hypotheses (H1 and H2), we conducted multiple linear regression analyses. Separate models were estimated for each sexual context (oral sex and vaginal intercourse) with the self-reported frequency of faking orgasm as the dependent variable. This allowed us to assess the unique contribution of each predictor while controlling for the shared variance.

Power analysis indicated that a sample of at least 127 participants was needed (see Section " Participants" for details); our final sample of 425 exceeded this threshold, ensuring sufficient statistical power.

The assumptions of normality, linearity, homoscedasticity, and multicollinearity were tested. All variables showed acceptable skewness and kurtosis (between −2 and +2), and Variance Inflation Factor (VIF) values were below 4. Residuals were normally distributed, as confirmed by Kolmogorov–Smirnov tests (ps > .05) and visual Q–Q plot inspection.

Although no formal power analysis exists for psychological network analysis, sample adequacy was assessed via bootstrap-based precision and stability metrics following best-practice guidelines (Epskamp et al., 2018).

## Results

### Descriptive statistics and correlations

Descriptive statistics are presented in Tables 1 and 2. Associations among variables were examined using network analysis, which estimates partial rather than zero-order correlations. As expected, the DERS subscales showed moderate to strong intercorrelations, particularly between impulse control difficulties and goal-directed behavior. The three sexual motivation subscales were also positively correlated, with the strongest association observed between coping-related motives and personal goal attainment. Faking orgasm subscales showed moderate associations with both emotion regulation difficulties and sexual motivations, supporting their inclusion in the subsequent network and regression analyses.

**Table 1.** Edge weights matrix between the variables from the network analyses.

	FOS Oral Sex subscales												
Variable	1	2	3	4	5	6	7	8	9	10	11	12	13
1. DERS Non-Acceptance	0.000	0.000	0.045	0.000	0.456	0.048	0.000	0.047	0.000	0.000	0.003	0.000	0.084
2. DERS Goals	0.000	0.000	0.456	−0.040	0.245	0.000	0.000	0.000	0.000	0.000	0.000	0.000	0.000
3. DERS Impulse	0.045	0.456	0.000	0.000	0.302	0.124	0.000	0.000	0.040	0.000	0.032	0.000	0.037
4. DERS Awareness	0.000	−0.040	0.000	0.000	0.000	0.429	0.000	−0.127	0.074	0.000	0.000	−0.049	0.000
5. DERS Strategies	0.456	0.245	0.302	0.000	0.000	0.150	0.000	0.000	0.000	0.000	0.104	0.000	0.000
6. DERS Clarity	0.048	0.000	0.124	0.429	0.150	0.000	0.025	0.000	0.136	0.000	0.006	0.000	0.108
7. YSEX?-HSF Personal Goal Attainment	0.000	0.000	0.000	0.000	0.000	0.025	0.000	0.082	0.363	0.000	0.000	0.234	0.000
8. YSEX?-HSF Relational Reasons	0.047	0.000	0.000	−0.127	0.000	0.000	0.082	0.000	0.192	0.144	0.000	0.124	0.000
8. YSEX?-HSF Sex as Coping	0.000	0.000	0.040	0.074	0.000	0.136	0.363	0.192	0.000	0.046	0.054	0.077	0.040
9. FOS OSAD	0.000	0.000	0.000	0.000	0.000	0.000	0.000	0.144	0.046	0.000	0.166	0.151	0.109
10. FOS OSIA	0.003	0.000	0.032	0.000	0.104	0.006	0.000	0.000	0.054	0.166	0.000	0.036	0.078
11. FOS OSEA	0.000	0.000	0.000	−0.049	0.000	0.000	0.234	0.124	0.077	0.151	0.036	0.000	0.058
12. FOS OSFD	0.084	0.000	0.037	0.000	0.000	0.108	0.000	0.000	0.040	0.109	0.078	0.058	0.000

*Note*: DERS = Difficulties in emotion regulation. YSEX?-HSF = Hungarian Short Form if Reasons of Having Sex Questionnaire. FOS = Faking Orgasm Scale for Women. OSAD = Oral Sex Altruistic Deceit; OSIA = Oral Sex Insecure Avoidance; OSEA = Oral Sex Elevated Arousal; OSFD = Oral Sex Fear of Dysfunction.

Regularized partial correlations have been reported. The edge weights were estimated using graphical LASSO with an EBIC tuning parameter of 0.5. Stronger edges reflect more robust associations after controlling for all other variables. All variables were standardized prior to the analysis.

**Table 2.** Edge weights matrix between the variables from the network analyses.

	FOS Sexual Intercourse subscales												
Variable	1	2	3	4	5	6	7	8	9	10	11	12	13
1. DERS Non-Acceptance	0.000	0.000	0.036	0.000	0.460	0.048	0.000	0.020	0.000	0.088	0.069	0.082	0.000
2. DERS Goals	0.000	0.000	0.458	−0.042	0.248	0.000	0.000	0.000	0.000	−0.039	0.028	0.000	−0.039
3. DERS Impulse	0.036	0.458	0.000	0.000	0.308	0.126	0.000	0.000	0.039	0.000	0.043	0.035	0.000
4. DERS Awareness	0.000	−0.042	0.000	0.000	0.000	0.432	0.000	−0.121	0.073	0.000	0.000	−0.069	0.000
5. DERS Strategies	0.460	0.248	0.308	0.000	0.000	0.155	0.000	0.000	0.000	−0.015	0.000	0.000	0.000
6. DERS Clarity	0.048	0.000	0.126	0.432	0.155	0.000	0.015	0.000	0.119	−0.027	0.073	0.000	0.106
7. YSEX?-HSF Personal Goal Attainment	0.000	0.000	0.000	0.000	0.000	0.015	0.000	0.093	0.367	0.000	0.029	0.165	0.036
8. YSEX?-HSF Relational Reasons	0.020	0.000	0.000	−0.121	0.000	0.000	0.093	0.000	0.220	0.109	0.000	0.158	−0.100
8. YSEX?-HSF Sex as Coping	0.000	0.000	0.039	0.073	0.000	0.119	0.367	0.220	0.000	0.066	0.051	0.015	0.189
9. FOS SIAD	0.088	−0.039	0.000	0.000	−0.015	−0.027	0.000	0.109	0.066	0.000	0.314	0.084	0.000
10. FOS SIFI	0.069	0.028	0.043	0.000	0.000	0.073	0.029	0.000	0.051	0.314	0.000	0.078	0.096
11. FOS SIEA	0.082	0.000	0.035	−0.069	0.000	0.000	0.165	0.158	0.015	0.084	0.078	0.000	0.016
12. FOS SISA	0.000	−0.039	0.000	0.000	0.000	0.106	0.036	−0.100	0.189	0.000	0.096	0.016	0.000

*Note*: DERS = Difficulties in emotion regulation. YSEX?-HSF = Hungarian Short Form if Reasons of Having Sex Questionnaire. FOS = Faking Orgasm Scale for Women. SIAD = Sexual Intercourse Altruistic Deceit; SIFI = Sexual Intercourse Fear and Insecurity; SIEA = Sexual Intercourse Elevated Arousal; SISA = Sexual Intercourse Sexual Adjournment.

The network structure was estimated using EBICglasso. All the values represent partial correlations. The visualized edges reflect nonzero associations between the nodes. Centrality and edge weight interpretations should be approached cautiously owing to their exploratory nature.

### Network analysis

**Figure 1.**
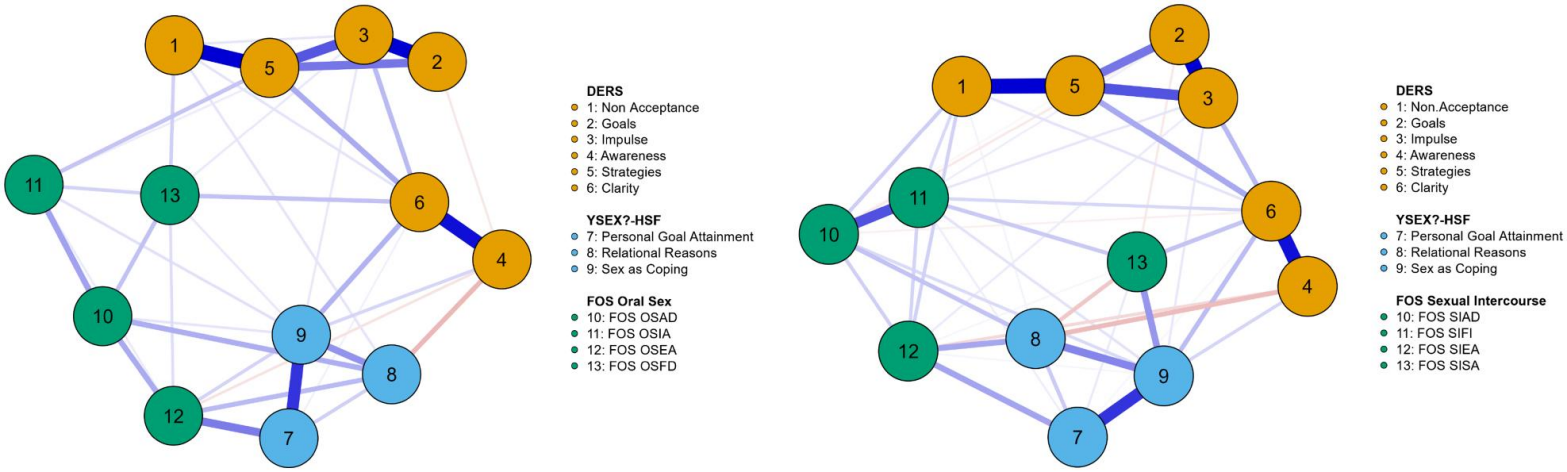
Visualization of the network of the variables included in our study separately for FOS Oral Sex subscales (left panel) and FOS Sexual Intercourse subscales (right panel). Blue lines represent positive associations and red lines represent negative associations. The thickness of the edge indicates the strength of the association. Nodes with stronger or more connections are closer together, and the most central nodes appear at the center. *Note*: This figure displays the regularized partial correlation network of the study variables. Abbreviations for the Difficulties in Emotion Regulation Scale (DERS) subscales: ‘Clarity’ refers to difficulties in identifying one’s emotional states, ‘Awareness’ refers to lack of emotional awareness, ‘Impulse’ refers to impulsivity-related difficulties in emotion regulation, ‘Goals’ indicates difficulties in goal-directed behavior under emotional distress, ‘Strategies’ represents limited access to effective emotion regulation strategies, and ‘Nonacceptance’ reflects nonacceptance of emotional responses. All other abbreviations correspond to the Faking Orgasm Scale for Women (FOS). OSAD = Oral Sex Altruistic Deceit; OSIA = Oral Sex Insecure Avoidance; OSEA = Oral Sex Elevated Arousal; OSFD = Oral Sex Fear of Dysfunction; SIAD = Sexual Intercourse Altruistic Deceit; SIFI = Sexual Intercourse Fear and Insecurity; SIEA = Sexual Intercourse Elevated Arousal; SISA = Sexual Intercourse Sexual Adjournment.

To explore the interrelations among psychological predictors and orgasm-faking tendencies, two network models were estimated: one for vaginal intercourse (FOS-SI subscales) and one for oral sex (FOS-OS subscales). Each model consisted of 13 nodes (six DERS subscales and three YSEX?-HSF subscales, and four FOS subscales). A fully connected network contains 78 edges; however, to obtain a parsimonious and interpretable structure, we applied the EBICglasso method with a tuning parameter of 0.5 (Epskamp et al., 2018). Figure 1 provides a graphical depiction of the two estimated networks, highlighting the partial correlations among emotion regulation difficulties, sexual motivations, and faking orgasm motives across contexts.

#### Oral sex context

The oral sex network (FOS-OS) resulted in 40 retained edges (see Table 1). As expected, the variables tended to cluster within instruments, but several cross-instrument associations emerged. Notably, the coping-related sexual motivation (“Sex as Coping”) showed small-to-moderate positive associations with multiple orgasm-faking subscales. Among the emotion regulation variables, impulse control difficulties and lack of emotional clarity were modestly associated with faking orgasm, motivated by fear of dysfunction (OSFD) and insecurity avoidance (OSIA).

These findings suggest that while orgasm faking during oral sex is less embedded in emotion regulation difficulties than in vaginal intercourse, it still reflects the relevant affective and motivational processes. The overall pattern points to a looser and more situationally variable structure.

#### Vaginal intercourse context

The vaginal intercourse network (FOS-SI) was denser, retaining 47 edges (see Table 2). Strong associations were observed between the DERS subscales (e.g., between difficulties in accessing regulation strategies and non-acceptance of emotions, or between impulse control problems and goal-directed behavior difficulties), reflecting well-known patterns of dysregulation.

Cross-instrument associations were more numerous and stronger than in the oral sex network. For example, faking orgasm due to partner reassurance (SIAD) was strongly linked to sex as coping and non-acceptance of emotions. Similarly, emotional clarity difficulties were connected to sexual adjournment faking (SISA), whereas personal goal attainment and relational motives were associated with arousal enhancement faking (SIEA).

These results indicate that orgasm faking during vaginal intercourse is more closely tied to emotional dysregulation and motivational factors than is faking during oral sex.

#### Centrality and summary

Across both networks, 13 edges were observed between the FOS and DERS subscales, and 7 between the FOS and YSEX?-HSF subscales. Within-network associations were also consistent with prior research, such as those between impulse control and goal-directed behavior (DERS) or between sex as coping and personal goal attainment (YSEX).

Centrality analyses (see Table 3) revealed that the most influential psychological predictors based on both strength and expected influence were impulse control difficulties, limited access to regulation strategies, lack of emotional clarity, and coping-related sexual motivation.

**Table 3.** Centrality indices of the variables from the network analyses.

	FOS Oral Sex subscales				FOS Sexual Intercourse subscales			
Variable	Betweenness	Closeness	Strength	Expected influence	Betweenness	Closeness	Strength	Expected influence
1. DERS Non-Acceptance	−0.685	−0.121	−0.462	−0.138	0.496	0.324	−0.306	0.261
2. DERS Goals	−0.947	−1.002	−0.202	−0.213	−0.956	−1.312	−0.034	−0.354
3. DERS Impulse	−0.161	−0.118	1.089	1.120	0.012	−0.393	0.962	1.054
4. DERS Awareness	−0.161	0.951	−0.296	−1.544	−0.149	0.810	−0.647	−1.464
5. DERS Strategies	1.279	1.175	2.056	1.904	0.981	0.979	1.692	1.411
6. DERS Clarity	2.588	2.214	1.040	1.081	2.594	2.040	1.242	1.056
7. YSEX?-HSF Personal Goal Attainment	−0.161	−0.449	−0.367	−0.060	−0.633	−0.692	−0.812	−0.055
8. YSEX?-HSF Relational Reasons	−0.292	0.070	−0.314	−0.920	−0.472	0.340	−0.213	−1.116
9. YSEX?-HSF Sex as Coping	0.755	0.548	1.018	1.063	0.819	0.821	1.443	1.355
10. FOS OSAD/SIAD	−0.161	−0.275	−0.752	−0.373	−0.794	−0.803	−0.621	−0.463
11. FOS OSIA/SIFI	−0.423	−0.632	−1.353	−0.861	−0.633	−1.453	−0.422	0.189
12. FOS OSEA/SIEA	−0.685	−1.268	−0.261	−0.326	−0.633	−0.362	−0.830	−0.516
13. FOS OSFD/SISA	−0.947	−1.091	−1.196	−0.733	−0.633	−0.299	−1.455	−1.359

*Note*: DERS = Difficulties in emotion regulation. YSEX?-HSF = Hungarian Short Form if Reasons of Having Sex Questionnaire. FOS = Faking Orgasm Scale for Women. OSAD = Oral Sex Altruistic Deceit; OSIA = Oral Sex Insecure Avoidance; OSEA = Oral Sex Elevated Arousal; OSFD = Oral Sex Fear of Dysfunction; SIAD = Sexual Intercourse Altruistic Deceit; SIFI = Sexual Intercourse Fear and Insecurity; SIEA = Sexual Intercourse Elevated Arousal; SISA = Sexual Intercourse Sexual Adjournment.

The centrality indices represent the relative importance of each variable in the network. Strength reflects the sum of the absolute edge weights, and expected influence considers the sign of the edges (positive or negative). CS = correlation stability coefficient based on 1,000 bootstraps. Values above 0.25 are acceptable; above 0.50 are considered good (Epskamp et al., 2018).

Taken together, network models support the idea that orgasm-faking behavior, particularly in the context of vaginal intercourse, is embedded within broader affective and motivational dynamics.

### Regression analyses

To formally test our hypotheses (H1 and H2), we conducted multiple linear regression analyses using Generalized Linear Models (GLMs) with the self-reported frequency of faking orgasm as the dependent variable. Predictors included the emotion regulation and sexual motivation subscales described in the Methods section.

#### Oral sex context

The models for oral sex (FOS-OS) explained between 5.4% and 14% of the variance (see Table 4). Key findings:

**Table 4.** Results of the GLM analysis: The presented values are standardized estimates (β).

		OSAD				OSIA			OSEA			OSFD		
		β/R^2^	df	t/F	*p*	β/R^2^	t/F	*p*	β/R^2^	t/F	*p*	β/R^2^	t/F	*p*
	Model	0.054	9, 141	1.95	0.050	0.066	2.17	0.028	0.140	3.70	< .001	0.093	2.70	0.006
DERS	Non Acceptance	0.017	141	0.15	0.88	0.090	0.82	0.41	0.061	0.57	0.56	*0.276*	*2.56*	*0.01*
	Goals	−0.054	141	−0.42	0.68	−0.017	−0.13	0.90	−0.068	−0.54	0.58	0.105	0.82	0.41
	Impulse	−0.030	141	−0.22	0.83	0.076	0.54	0.58	0.002	0.02	0.98	0.129	0.94	0.34
	Awareness	−0.090	141	−0.87	0.38	−0.126	−1.24	0.22	−0.179	−1.83	0.07	−0.020	−0.20	0.84
	Strategies	0.077	141	0.52	0.60	0.154	1.05	0.29	−0.009	−0.07	0.94	−0.268	−1.85	0.07
	Clarity	0.057	141	0.50	0.62	0.081	0.71	0.47	0.109	1.00	0.31	0.179	1.59	0.11
YSEX?-HSF	Personal Goal Attainment	−0.098	141	−1.03	0.31	−0.023	−0.24	0.81	*0.206*	*2.26*	*0.03*	−0.066	−0.71	0.48
	Relational Reasons	*0.262*	*141*	*2.76*	*0.01*	−0.075	−0.79	0.42	*0.178*	*1.98*	*0.05*	−0.059	−0.63	0.52
	Sex as Coping	0.150	141	1.47	0.14	0.163	1.61	0.11	0.121	1.25	0.21	0.172	1.72	0.09
		SIAD				SIFI			SIEA			SISA		
		β/R^2^	df	t/F	*p*	β/R^2^	t/F	*p*	β/R^2^	t/F	*p*	β/R^2^	t/F	*p*
	Model	0.115	9, 198	3.89	< .001	0.121	4.18	< .001	0.168	5.64	< .001	0.133	4.53	< .001
DERS	Non Acceptance	*0.344*	*198*	*3.67*	*< .001*	0.172	1.84	0.07	*0.226*	*2.48*	*0.01*	0.061	0.65	0.51
	Goals	−0.081	198	−0.81	0.42	0.067	0.67	0.50	0.022	0.22	0.82	−0.136	−1.36	0.17
	Impulse	0.089	198	0.78	0.43	0.081	0.70	0.48	0.195	1.75	0.08	0.067	0.59	0.55
	Awareness	0.083	198	0.98	0.32	−0.073	−0.86	0.38	−0.102	−1.23	0.22	−0.123	−1.45	0.15
	Strategies	−0.202	198	−1.72	0.09	−0.063	−0.53	0.59	−0.212	−1.85	0.07	−0.049	−0.42	0.67
	Clarity	−0.110	198	−1.18	0.24	0.153	1.64	0.10	−0.025	−0.27	0.78	*0.233*	*2.50*	*0.01*
YSEX?-HSF	Personal Goal Attainment	−0.067	198	−0.88	0.38	0.046	0.61	0.54	*0.151*	*2.02*	*0.04*	0.075	0.99	0.32
	Relational Reasons	*0.172*	*198*	*2.28*	*0.02*	0.026	0.34	0.73	*0.178*	*2.42*	*0.02*	*−0.237*	*−3.16*	*<.01*
	Sex as Coping	*0.182*	*198*	*2.19*	*0.03*	0.161	1.93	0.05	0.094	1.16	0.25	*0.306*	*3.71*	*< .001*

*Note*: OSAD = Oral Sex Altruistic Deceit; OSIA = Oral Sex Insecure Avoidance; OSEA = Oral Sex Elevated Arousal; OSFD = Oral Sex Fear of Dysfunction; SIAD = Sexual Intercourse Altruistic Deceit; SIFI = Sexual Intercourse Fear and Insecurity; SIEA = Sexual Intercourse Elevated Arousal; SISA = Sexual Intercourse Sexual Adjournment. Standardized regression coefficients (β) were calculated. The adjusted R^2^ values are presented for each model. *Italicized values indicate statistically significant predictors (p < .05)*.

- Partner assurance faking (OSAD) was positively predicted by relationship-focused sexual motivation.
- Insecurity avoidance faking (OSIA) was not significantly predicted by any individual variable, although the model was statistically significant.
- Elevated arousal faking (OSEA) was positively predicted by both personal goal attainment and relationship-focused motives.
- Dysfunction-avoidance faking (OSFD) was positively predicted by non-acceptance of emotions.

These results suggest that, while emotional and motivational factors play a role in faking orgasm during oral sex, the strength and consistency of these predictors are somewhat limited.

#### Vaginal intercourse context

The models for vaginal intercourse (FOS-SI) explained 11.5%–16.8% of the variance (see Table 4). Several robust patterns have emerged in this regard.

- Partner assurance faking (SIAD) was positively predicted by non-acceptance of emotions, relationship-focused motives, and coping-related sexual motivation.
- Insecurity avoidance faking (SIFI) model was statistically significant, although no single predictor emerged as significant.
- Elevated arousal faking (SIEA) was significantly predicted by non-acceptance of emotions, personal goal attainment, and relational motives.
- Sexual adjournment faking (SISA) was positively predicted by lack of emotional clarity and sex as coping, and negatively predicted by relational motives.

These findings support both hypotheses: Difficulties in emotion regulation and specific sexual motivations, particularly coping-related and partner-oriented motives, are reliable predictors of faking orgasm, especially during vaginal intercourse.

### Summary of findings

The present study examined the psychological predictors of faking orgasm in two sexual contexts, oral sex and vaginal intercourse, using both network analysis and multiple regression. These findings provide converging evidence across the analytic strategies.

As hypothesized, difficulties in emotion regulation—particularly non-acceptance of emotions, impulse control problems, and lack of emotional clarity—were associated with increased tendencies to fake orgasm (H1). Similarly, coping-related, self-focused, and partner-oriented sexual motivations were positively related to faking orgasm, with coping-related motives emerging as particularly influential (H2).

Although both sexual contexts were associated with emotional and motivational factors, the patterns were more pronounced and consistent in vaginal intercourse. This suggests that a faking orgasm in this context may be more tightly embedded within broader affective and relational dynamics.

Together, these results support a multidimensional understanding of faking orgasm, in which emotional dysregulation and strategic sexual motivation interact to shape women’s sexual behavior across contexts.

## Discussion

This study investigated the psychological underpinnings of faking orgasm by simultaneously examining emotion regulation difficulties and sexual motivations across two sexual contexts: oral sex and vaginal intercourse. Using both network analysis and multiple regression, the findings highlighted that orgasm-faking behaviors are significantly associated with specific emotion regulation deficits (e.g., non-acceptance of emotions and emotional clarity) and strategic sexual motives, particularly coping-related reasons. These results extend prior research by integrating two often-separately studied domains—emotional functioning and sexual motivation—into a unified framework.

In what follows, we first discuss the theoretical implications of our findings in light of the existing literature on emotion regulation and sexual behavior. We then considered the differences between the sexual contexts and their possible interpretations. Finally, we reflect on the methodological limitations of our study and outline the directions for future research.

### Theoretical implications

The present findings deepen our understanding of how difficulties in emotion regulation and sexual motivations intersect in shaping women’s orgasm-faking behavior. Consistent with Cooper’s (2014) cluster analysis findings, our results support the notion that faking orgasm may serve affect-regulatory purposes, particularly when women face emotional discomfort or relational demands. Specifically, difficulties in the non-acceptance of emotions, impulse control, and emotional clarity emerged as the core emotional traits associated with orgasm-faking, suggesting that faking may function as a compensatory strategy in emotionally charged sexual encounters.

These results align with prior work on affective regulation in sexual behavior, which has shown that women often engage in sex not solely for physical pleasure but also to manage negative emotional states (Impett et al., 2005; Bőthe et al., 2020). Meston and Buss (2007) emphasized the psychological complexity of sexual motivations, highlighting that many individuals pursue sex to meet psychological or relational needs. Our findings extend this by showing that when these motives—particularly coping-related ones—are paired with emotional dysregulation, they may lead to strategic sexual behaviors, such as faking orgasm.

The association between coping-related motives and faking orgasm resonates with theories that frame sexual behavior as embedded in sociocultural scripts and expectations (Frith, 2018; Lehmiller, 2023). For women, these scripts often prioritize partner satisfaction and relational harmony, potentially reinforcing the tendency to simulate orgasm when emotional or relational pressure is high (Thomas et al., 2017). This strategic accommodation can be seen as a form of avoidant coping, helping women manage emotionally uncomfortable or ambivalent sexual experiences (Jonason, 2019).

Notably, our results echo earlier findings linking shame, self-blame, and negative body image to reduced orgasmic functioning and increased faking (Nobre & Pinto-Gouveia, 2008; Rowland et al., 2018; Tavares et al., 2018). Taken together, these patterns suggest that faking orgasm is not merely a relational tactic but may reflect a deeper emotional regulatory mechanism that is shaped by individual vulnerabilities and broader sociocultural expectations.

This interpretation is further supported by our network analysis, which identified high centrality for both coping-related sexual motivation and difficulties in emotion regulation. These findings contribute to a growing body of research suggesting that sexual behavior, particularly in women, cannot be fully understood without accounting for the emotional and motivational dynamics that underlie it. These results also resonate with the sexual script theory, which posits that sexual behavior is guided by culturally shared narratives that prescribe appropriate roles, goals, and outcomes for each gender (Frith and Kitzinger, 2001; Simon and Gagnon, 1986). In this view, faking orgasm can be understood as an enactment of normative scripts that emphasize female responsibility for male satisfaction, reinforcing expectations around performance and relational harmony.

### Sexual contexts: Oral vs. vaginal intercourse

The present study differentiated between two sexual contexts, oral sex and vaginal intercourse, to explore whether psychological predictors of orgasm faking operate similarly across situations. This distinction, originally emphasized by Cooper (2014), proved to be theoretically and empirically meaningful. Although both contexts showed associations between faking orgasm and psychological variables, the patterns differed in strength and specificity.

Faking an orgasm during vaginal intercourse was more consistently and strongly associated with emotion regulation difficulties and coping-related sexual motivations. This finding suggests that vaginal intercourse may be a more emotionally and relationally loaded context for many women, where the stakes of sexual performance and partner satisfaction are perceived to be higher. Such interpretations are consistent with sexual script theory, which posits that heterosexual intercourse carries culturally embedded expectations—particularly for women—to engage in “successful” sex that includes a visible orgasmic response (Frith, 2018; Lehmiller, 2023). Faking an orgasm in this context may thus serve as a socially scripted behavior to meet perceived relational obligations and avoid disrupting partner dynamics (Thomas et al., 2017).

In contrast, faking orgasm during oral sex is less tightly embedded in broader emotional and motivational profiles. Although meaningful associations were still found, especially with coping-related motives and the non-acceptance of emotions, the overall structure was sparser. This may reflect the lower normative pressure associated with orgasm during oral sex as well as differing levels of partner involvement or expectations (Herbenick et al., 2019). Prior research has also shown that women are more likely to achieve orgasm during oral sex than during intercourse (Armstrong et al., 2012), possibly reducing their need or motivation to fake.

These context-specific findings highlight the importance of distinguishing sexual behaviors not only by type but also by their emotional and relational embeddings. The results suggest that faking orgasm may serve different functions across contexts, more as a strategic affective regulation mechanism in vaginal intercourse and potentially more situational or performance-oriented during oral sex. Future research should explore how emotional and motivational dynamics vary across sexual acts, partner types, and relational settings.

These patterns may also reflect the influence of heteronormative models, which frame heterosexual intercourse as prototypical or idealized sexual activity (Jackson, 2006). In such models, women may feel pressured to conform to the expectation of orgasm during vaginal intercourse, even when the experience is not pleasurable or emotionally attuned, leading to strategic behaviors, such as faking orgasm to maintain normative appearances.

### Clinical and social relevance

These findings have important implications for both clinical practice and the public discourse on female sexuality. Understanding faking orgasm as a possible indicator of emotion regulation difficulties and affect-driven sexual motivation may assist therapists in identifying the maladaptive coping strategies that manifest in sexual contexts. For example, clinicians working with women reporting low sexual satisfaction or sexual communication difficulties may consider exploring the emotional and motivational underpinnings of orgasm-faking as part of a broader affective and relational assessment.

Coping-related sexual motivations, such as having sex to reduce anxiety, avoid conflict, or maintain emotional closeness, may not always be consciously acknowledged by clients. Yet, as this study suggests, these motivations can interact with emotional dysregulation to foster strategic sexual behaviors, such as faking orgasm, which may initially preserve relational harmony but ultimately perpetuate sexual dissatisfaction or emotional disconnection. Recognizing these patterns may support more nuanced therapeutic work, especially in couples and sex therapy settings.

On a broader level, these results highlight the role of cultural narratives and social expectations in shaping women’s sexual behaviors. When women internalize norms that prioritize their partners’ satisfaction or emotional stability over their own authenticity or pleasure, they may feel compelled to simulate orgasms as a means of meeting those expectations. Educational efforts that normalize a diversity of orgasmic experiences and promote open sexual communication could help reduce the perceived need for fake orgasm and foster healthier sexual dynamics.

The identification of key emotional traits, such as impulse control difficulties, limited access to emotion regulation strategies, and poor emotional clarity, as central predictors of faking behavior also underscores the need for more integrated psychological approaches to sexuality. Emotional literacy, mindfulness-based interventions, and emotion-focused therapy techniques may be useful in helping individuals improve their emotional regulation capacities and develop more satisfying sexual relationships.

In sum, these findings support a growing view of sexuality as deeply intertwined with psychological and emotional functioning and suggest that faking orgasm can serve as a behavioral lens through which deeper emotional needs, vulnerabilities, or unspoken relational scripts are expressed.

### Limitations and future directions

Although this study provides novel insights into the psychological underpinnings of faking orgasm, several limitations must be acknowledged. First, its cross-sectional and correlational design precludes causal inferences. Although the network analysis revealed meaningful associations and centrality patterns, it could not determine the directionality of the effects. Future research using longitudinal or experimental designs is needed to clarify how emotion regulation and sexual motivation dynamically influence orgasm-faking behavior over time or across relational transitions.

Second, the use of self-report measures may have introduced biases related to social desirability, memory recall, or limited introspective access, particularly in relation to sexual motivation and affective regulation. While retrospective self-reports can meaningfully capture aspects of sexual behavior (Cooper, 2014; Muehlenhard & Shippee, 2010), they may not fully convey the lived emotional nuances involved in orgasm-faking. Although a growing number of qualitative studies have examined women’s experiences of faking orgasm, few have directly explored this behavior in relation to emotion regulation difficulties and coping-related sexual motives, which are central to the present study. Future qualitative work focused specifically on these psychological dimensions could offer richer insights into the affective functions of orgasm-faking in everyday sexual contexts.

Third, although our study focused on the frequency of and motivations for faking orgasm, we did not assess whether participants were generally able to achieve orgasm. Since faking orgasm is conceptually distinct from orgasmic difficulty, the absence of a measure assessing orgasmic functioning limits our ability to contextualize the motivations behind faking. Future studies should include direct assessments of orgasm frequency and consistency to better distinguish between strategic faking and compensatory responses to sexual dysfunction.

Fourth, although we assessed six facets of emotion regulation and three sexual motivation dimensions, other potentially relevant psychological constructs, such as attachment style, self-esteem, shame, and sexual assertiveness, were not included. Integrating these variables in future models could enhance explanatory power and contribute to a more comprehensive understanding of why and when women fake orgasms.

Fifth, the sample was limited to self-identified women who were recruited through convenience sampling in Hungary. The predominance of young heterosexual participants may limit the generalizability of the findings to more diverse populations. Cultural norms around sexuality and emotional expression may shape both the likelihood and meaning of orgasm faking; therefore, cross-cultural and LGBTQ+-inclusive studies are urgently needed.

Sixth, although we collected basic demographic data (age, relationship status, and sexual orientation), we did not include these variables as covariates in the main analysis. This decision was informed by our study’s focus on psychological predictors, and preliminary analyses indicated that these demographic factors were not significantly associated with outcome variables. However, we acknowledge that such variables may still interact with emotional and motivational patterns in more complex ways, particularly in light of heteronormative social scripts that shape sexual expectations and behaviors. The omission of these covariates limits the depth of interpretation and may obscure important subgroup differences. Future studies should explicitly examine the moderating role of demographic and relational variables in shaping orgasm-faking tendencies, and consider their inclusion in predictive models.

Although our sample included a notable proportion of participants who did not identify themselves as exclusively heterosexual, we did not conduct subgroup analyses based on sexual orientation. This decision reflects both the study’s primary focus on psychological predictors and the need to maintain a clear and coherent analytical framework. Including sexual orientation as an additional explanatory variable would have required a distinct conceptual and methodological designideally supported by a dedicated sample and instruments tailored to the experiences of sexual minority groups. Nevertheless, we acknowledge that valuable insights could be gained by exploring how sexual orientation intersects with emotional and motivational dynamics in orgasm-faking behavior. Future studies should pursue this direction to better understand how sexual identity shapes the affective and strategic dimensions of sexual functioning.

Finally, our use of “vaginal intercourse” and “oral sex” as context categories followed prior literature (Cooper, 2014), but additional contextual distinctions, such as sexual initiation, partner type, and relational satisfaction, may offer further nuance. Future research should aim to disentangle these layers and examine how they interact with the emotional and motivational patterns.

Despite these limitations, this study contributes to a growing body of research highlighting the psychological complexity of women’s sexual behaviors. By foregrounding the roles of emotion regulation and coping-related sexual motives, our findings open new avenues for both theoretical refinement and applied interventions in clinical and educational settings.

## Data Availability

The data that support the findings of this study are available at: https://osf.io/eyrwd/?view_only=b0d36d6df9fa4bdfa9c2d6883b5dfeaf.
